# A miR‐206 regulated gene landscape enhances mammary epithelial differentiation

**DOI:** 10.1002/jcp.28789

**Published:** 2019-05-08

**Authors:** Jun Wang, Eylem Aydoğdu, Srijita Mukhopadhyay, Luisa A. Helguero, Cecilia Williams

**Affiliations:** ^1^ Department of Biology and Biochemistry, Center for Nuclear Receptors and Cell Signaling University of Houston Texas; ^2^ Department of Protein Science, KTH Royal Institute of Technology Science for Life Laboratories Stockholm Sweden; ^3^ Department of Plant Biotechnology and Bioinformatics Ghent University Ghent Belgium; ^4^ VIB Center for Plant Systems Biology Ghent Belgium; ^5^ Department of Medical Sciences Institute of Biomedicine University of Aveiro Aveiro Portugal

**Keywords:** cell cycle, cell differentiation, mammary gland, miR‐206, tumor suppressor

## Abstract

miR‐206 is known to suppress breast cancer. However, while it is expressed in mammary stem cells, its function in such nontumor cells is not well understood. Here, we explore the role of miR‐206 in undifferentiated, stem‐like mammary cells using the murine mammary differentiation model HC11, genome‐wide gene expression analysis, and functional assays. We describe the miR‐206‐regulated gene landscape and propose a network whereby miR‐206 suppresses tumor development. We functionally demonstrate that miR‐206 in nontumor stem‐like cells induces a G1–S cell cycle arrest, and reduces colony formation and epithelial‐to‐mesenchymal transition markers. Finally, we show that addition of miR‐206 accelerates the mammary differentiation process along with related accumulation of lipids. We conclude that miR‐206 impacts a network of signaling pathways, and acts as a regulator of proliferation, stemness, and mammary cell differentiation in nontumor stem‐like mammary cells. Our study provides a broad insight into the breast cancer suppressive functions of miR‐206.

## 1 INTRODUCTION

The mammary gland develops from mammary stem cells. Bipotent mammary stem cells remain in the adult mammary gland, they drive its development during puberty and the stem cells expand during pregnancy. These cells can self‐renew as well as differentiate into both luminal and myoepithelial cells. The stem cells are also immortal and can accumulate mutations during their extended lifetime. As such, they have a higher risk of developing into cancer. Stem cells and cancer stem cells share numerous signaling pathways, and the molecular mechanisms that control stemness are critical in cancer (Nguyen, Vanner, Dirks, & Eaves, 2012). The idea of targeting these mechanisms for novel cancer therapeutics is an attractive approach, and these pathways is therefore of specific interest to understand.

MicroRNAs (miRNAs) have key roles both in stem cells and cancer cells (Vahidian et al., 2018). The miR‐200a, b, and c members of the miR‐200 family have been identified as tumor suppressor miRNAs. They can repress stem cell abilities and epithelial‐to‐mesenchymal transition (EMT), and their downregulation in breast cancer stem cells allows their expansion and related resistance to therapeutics (Iliopoulos et al., 2010; Shimono et al., 2009). miR‐206 has also been described as a tumor suppressor miRNA that is downregulated in breast cancer (Li, Hong, & Yu, 2013). We and others have found that addition of miR‐206 inhibits cell proliferation of triple‐negative breast cancer (TNBC) cells (Hesari et al., 2018; Wang et al., 2014). We have also demonstrated that miR‐206 reduces the migratory potential of TNBC cells through repression of Coronin 1C (CORO1C; Wang et al., 2014). The miR‐206‐mediated repression of CORO1C enables a change in actin skeleton and cell morphology which reduces migration, but CORO1C does not affect proliferation (Wang et al., 2014). miR‐206 has been shown to control embryonic mammary development by repressing the estrogen receptor (ER/ESR1) and modifying Wnt signaling (Adams, Furneaux, & White, 2007; [Link] Yoon, Cho, Kim, & Jung). Further, overexpression of miR‐206 was recently shown to increase fat tissue and reduce branching morphogenesis in BRCA1‐depleted mouse mammary gland (Wronski et al., 2016). Clearly, miR‐206 has roles during mammary development. We do not know, however, exactly how miR‐206 impacts the gene expression in nontumor mammary cells, nor do we understand the mechanism by which it impacts proliferation, fat accumulation, and breast cancer development. To understand this, it is important to understand its activities in nontumor cells.

We have previously studied mammary differentiation and stem cell‐related mechanisms using the murine cell line model HC11. This cell line (descended from COMMA‐1D) can be cultured in an undifferentiated, proliferating, mammary stem cell‐like stage that exhibits properties specific for mammary stem cells (Ball, Friis, Schoenenberger, Doppler, & Groner, 1988; Merlo et al., 1996). The cell line further allows for induction of functional differentiation by removal of growth factors (EGF) and addition of lactogenic hormones (dexamethasone, insulin, and prolactin). Functional differentiated cells produce the milk protein β‐casein in vitro (Ball et al., 1988; Merlo et al., 1996). We have previously found similarities between the undifferentiated transcriptome of HC11 cells and that of poor‐prognosis TNBC cells (Aydoğdu et al., 2012; Monteiro et al., 2017; Williams, Helguero, Edvardsson, Haldosen, & Gustafsson, 2009). This includes regulations of miR‐200 members miR‐200a and b which are barely expressed in the stem cell‐like stage of HC11 cells but strongly upregulated during differentiation (Aydoğdu et al., 2012). Using this model system, we revealed that miR‐200a represses the Eph receptor A2 (Epha2) in HC11 cells and in human TNBC cells (Tsouko, Wang, Frigo, Aydoğdu, & Williams, 2015). EPHA2 is a target of interest in breast cancer and a potential prognostic biomarker. Although both miR‐200a/b and miR‐206 are characterized as breast tumor suppressor miRNAs, they are differently regulated during differentiation: miR‐200a/b is absent in stem cells or stem‐like HC11 cells but strongly upregulated during differentiation, whereas miR‐206 is enhanced in the stem‐like stage (Aydoğdu et al., 2012). In the present study, we aimed to systematically explore the role of miR‐206 in the nontumor, undifferentiated mammary stem‐like cells, including its overall regulatory role at the transcriptome level and corresponding functional impact. Understanding the function of miR‐206 in normal mammary cells can help us better understand how it can suppress tumor development.

## 2 MATERIALS AND METHODS

### 2.1 Cell culture and induction of differentiation

Murine mammary epithelial cell line HC11 was maintained in RPMI 1640 medium (Gibco, Grand Island, NY) supplemented with 10% fetal bovine serum (FBS), 5 µg/ml insulin, 10 ng/ml epidermal growth factor (EGF), and 50 µg/ml gentamicin (all from Sigma, Saint Louis, MO). Proliferating cells (P) were obtained under these growth conditions. Cells of predifferentiated (PD) and fully differentiated (FD) stages were acquired sequentially after induction of differentiation as previously described (Williams et al., 2009). In short, pre‐differentiation was induced by removal of EGF and reducing FBS to 2% for 48 hr, whereas full differentiation was accomplished by the subsequent addition of 100 nM dexamethasone and 1 µg/ml ovine prolactin for 72 hr.

### 2.2 miRNA mimic transfections

Human and mouse miR‐206 sequence are identical and we used the miRIDIAN miR‐206 mimic (MIMAT0000239: UGG AAU GUA AGG AAG UGU GUG G) along with negative control sequence (NC‐mimic) based on *Caenorhabditis elegans* miR‐67 (cel‐miR‐67: UCA CAA CCU CCU AGA AAG AGU AGA), which has minimal sequence identity in human, mouse, and rat. Both mimics were purchased from Dharmacon (Pittsburgh, PA) and were transfected at a final concentration of 30 nM using DharmaFECT1 (Dharmacon). During HC11 differentiation experiments, sequential transfection was done as outlined in Figure S1.

### 2.3 Animals and mammary gland tissue

Mammary gland from 2‐month old virgin, 10‐day pregnant, and 6‐day lactation mice were collected previously, as described in (Williams et al., 2009).

### 2.4 RNA extraction, complementary DNA synthesis, and quantitative polymerase chain reaction

Total RNA, including the miRNA population, was extracted using TRIzol (Invitrogen, Grand Island, NY) and miRNeasy kits (QIAGEN, Valencia, CA) according to the manufacturer's protocol. Complementary DNA (cDNA) synthesis and quantitative polymerase chain reaction (qPCR) for mRNA and miRNA were performed as previously described (Aydoğdu et al., 2012). Briefly, 1 µg of total RNA was subject to cDNA synthesis using SuperScript III reverse transcriptase (Invitrogen) and 10 ng of cDNA was used as template for qPCR with Fast SYBR Green SuperMix (Life Technologies, Grand Island, NY). The18S gene and/or 36B4 was used as a reference control. For miRNA, 100 ng total RNA was subjected to cDNA synthesis and subsequent qPCR using the TaqMan small RNA assay kit (Life Technologies). U6 was used as reference control.

### 2.5 Microarray experiment

Undifferentiated HC11 cells were transfected with miR‐206 mimic or negative control, twice in 24 hr intervals, and microarray analysis was performed 24 hr after final transfection. RNA isolated after treatments were analyzed in biological and technical duplicates. Spotted 70‐mer arrays covering 36,000 genes and variants (full protein‐coding genome) were used (Human Genome OpArray, Microarray Inc., Huntsville, AL) as previously described (Edvardsson, Ström, Jonsson, Gustafsson, & Williams, 2011; Simon, Mesmar, Helguero, & Williams, 2017). Slides were hybridized using a dye‐swap design and scanned using GenePix 4300A microarray scanner (Molecular Devices, Sunnyvale, CA).

### 2.6 Western blot analysis

Cells were washed with phophate buffered saline (PBS) and lysed in radioimmunoprecipitation assay buffer. Protein was quantified by Pierce 660 nm protein assay kit (Thermo Fisher Scientific, Waltham, MA). Around 30 µg of total protein were resolved on a 10% sodium dodecyl sulfate polyacrylamide gel electrophoresis, and transferred to nitrocellulose membranes according to standard procedures. Membranes were then blocked in 5% milk (in TBST) and incubated with primary antibodies against Melk (rabbit, polyclonal; catalog number 2274; 1:800 dilution; Cell Signaling Technology, Danvers, MA), PARP (1:1000 dilution; Cell Signaling Technology), Caspase3 (1:500 dilution; Cell Signaling Technology), and β‐actin as loading control (1:6000 dilution; Sigma‐Aldrich), overnight. Membranes were then incubated with corresponding horseradish peroxidase‐linked secondary antibody and visualized using Pierce ECL western blot analysis substrate (Thermo Fisher Scientific) according to the manufacturer's protocol.

### 2.7 Cell counting

HC11 cells were transfected with miR‐206 mimic and corresponding negative control for 48 hr as described above, trypsinized and stained with trypan blue. Viable cells were counted using Countess automatic cell counter (Invitrogen). Experiments were repeated in three independent assays, each performed in triplicate.

### 2.8 BrdU staining

Synchronized cells (0.5% BSA, 48 hr) were transfected with miR‐206 mimic and corresponding negative control. After 48 hr, BrdU (30 µM) was added for 60 min. Cells were fixed (EtOH, 70%) and washed (PBS; 2 N HCl/Triton X‐100, tetraborate) and incubated with FITC‐conjugated BrdU antibody (BD Biosciences, San Jose, CA) for 30 min, followed by FACS analysis.

### 2.9 Cell cycle analysis

Synchronized cells (0.5% BSA, 48 hr) were transfected with miR‐206 mimic and corresponding negative control. After 48 hr, cells were fixed (EtOH, 70%) and stained with propidium iodide (PI; 50 µg/ml; Sigma‐Aldrich). The cell cycle distribution of G0/G1, S, and G2/M phase was determined using BD FACSAria III (BD Biosciences) and data was analyzed using FlowJo V10.

### 2.10 Colony formation assay

Cells were transfected with miR‐206 mimic and corresponding negative control in six‐well plates (100,000 cell/well). After 24 hr, cells were trypsinized, washed (PBS), and diluted with complete medium (330 cell/ml) and seeded (1000 cells per 50 mm Petri dish) in triplicates. Colonies were allowed to form for 10 days, with fresh complete medium every second day. Cells were fixed with methanol: acetic acid (2:1) and stained with crystal violet (0.5%, in 25% methanol). Colonies were counted using the ImageJ software. The experiments were replicated three times.

### 2.11 Mammosphere formation assay

Cells were transfected with miR‐206 mimics or negative control, incubated (24 hr), and diluted in MammoCult human medium, supplemented with MammoCult proliferation supplements (Stemcell Technologies, BC, Canada) in a 1:10 ratio, 4 μg/ml (0.0004%) heparin solution, 0.48 μg/ml hydrocortisone, and 1% PEST. Cells were seeded in low‐adherent six‐well plates (10,000 cells/well) with 2 ml of final volume. Cells were incubated (5% CO_2_; 37°C) for 7 days to allow primary mammospheres to form. Plates were scanned in 2400 dpi resolution using GelCount, version 1.1.2.0 (Oxford Optronix Ltd, Milton, UK). For secondary cultures, cells from the primary culture were rinsed twice with PBS, brought to single‐cell suspension using Trypsin–EDTA treatment, and mammosphere assays conducted following the miR‐206 mimics or control transfection as described above, but with 5000 cells/well. Plates were scanned and images of the mammospheres were analyzed as above. For primary mammosphere assays, three experiment were performed using HC11 cells of three different passages, each in technical triplicates. For secondary mammosphere assays, three experiment were performed using HC11 cells of three different passages, one in technical triplicates and two without technical replicates.

### 2.12 CD24/CD44 FACS analysis

Human TNBC MDA‐MB‐231 and SUM159 cells were transfected with miR‐206 mimic or negative control (48 hr), collected and incubated with CD24‐FITC and CD44‐APC (both from BD Biosciences) on ice for 15 min. Cells were then resuspended in 0.5 µg/ml PI and analyzed on BD FACSAria III.

### 2.13 Oil Red O staining

HC11 cells were cultured under differentiation conditions and transfected with miR‐206 or negative control once at each stage of the differentiation process (total three times; Figure S1). Cells from each stage were washed twice (ice‐cold PBS), fixed (10% formalin, 1 hr room temperature), rinsed (distilled water), submerged in 60% isopropanol (5 min), and stained with Oil Red O (M312512; Thermo Fisher Scientific ) for 10 min. Pictures were taken under microscope (Olympus) and Oil Red O was eluted by adding 100% isopropanol for 10 min, and optical density (wavelength 500 nm) measured for quantification.

**Figure 1:**
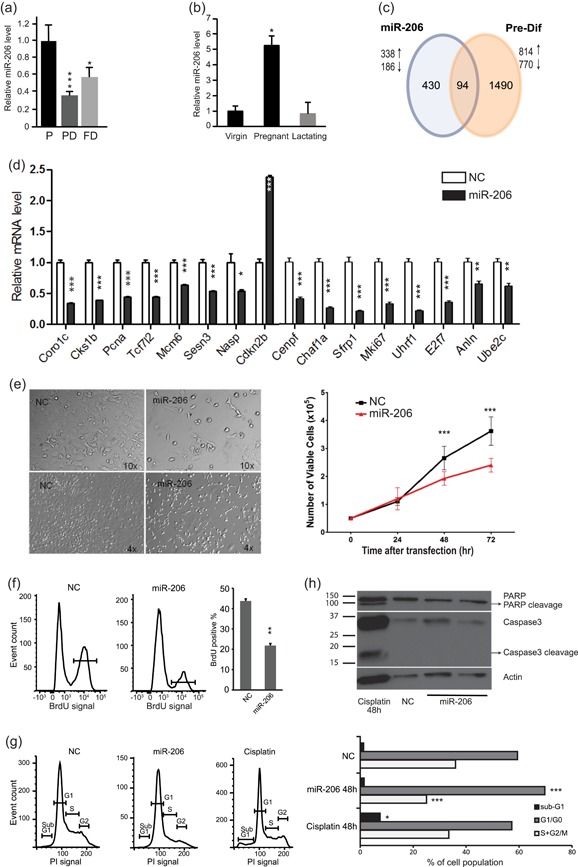
miR‐206 is upregulated in undifferentiated mammary cells where it modifies the transcriptome and controls cell proliferation. (a) miR‐206 levels are increased in the stem‐cell like stage of HC11 cells and reduced during differentiation. Analysis by TaqMan small RNA qPCR assay. (P) indicates proliferative stem‐like stage, (PD) predifferentiated stage, and (FD) functionally differentiated. (b) miR‐206 is upregulated in the mid‐pregnant mammary gland compared to the mammary gland of virgin or lactating mice. (c) The HC11 transcriptome is regulated by miR‐206 per microarray analysis (left circle). A proportion of the genes are also changed during HC11 differentiation (intersection). Genes regulated during HC11 cell differentiation (right circle) were previously determined by microarray analysis comparing undifferentiated stage with predifferentiated stage. (d) Numerous cell cycle‐related genes were identified as regulated by miR‐206 in the microarray analysis and are corroborated by qPCR. (e) miR‐206 (30 nM) blocks cell viability of undifferentiated HC11 cells, illustrated by a representative photo (left) and by cell counting of viable cells (right). (f) miR‐206 blocks DNA synthesis during the S cell cycle phase of HC11 cells, measured by BrdU incorporation. (g) miR‐206 induces a G1–S cell cycle arrest in HC11 cells, measured using the DNA‐specific PI dye, followed by FACS analysis. The G1/G0 population is significantly increased following miR‐206 mimic treatment, while the S ‐ G2/M population is correspondingly decreased, as visualized (left) and quantified (right). A sub‐G1 peak appeared after cisplatin treatment (positive control for apoptosis) but not after miR‐206 mimic. H) miR‐206 does not induce apoptosis in HC11 cells. The cleavage of apoptotic markers PARP and Caspase 3 (Casp3) was measured in miR‐206 mimic transfected cells, with cisplatin‐treated cells as positive control. Cleavage was detected after cisplatin treatment (48 hr) but not after miR‐206 treatment. Histogram bars indicate mean, +/− standard deviation (*SD*) and Student's unpaired two‐tailed *t* test was applied (a, d–g), and in (b) +/− standard error of the mean (*SEM*) and ANOVA. Significance is indicated by *(*p* < .05) **(*p* < .01), ***(*p* < .001). NC: negative control mimic. ANOVA: analysis of variance; FACS: fluorescence‐activated cell sorting; qPCR: quantitative polymerase chain reaction [Color figure can be viewed at wileyonlinelibrary.com]

### 2.14 Bioinformatic and statistical analyses

For the microarray analyses, GenePix Pro 6.0 software (Molecular Devices), R software (version 3.3.2) and the limma package, were used to filter and normalize data, perform dye‐swaps, generate M‐values [log2(fold change)] for each slide, and perform statistical analyses (Bonferroni correction), as previously described (Edvardsson et al., 2011; Simon et al., 2017). Genes were considered differentially expressed if *p *< 0.05 and |> 0.8. A less restrictive selection with *p* < 0.1 and | > 0.6 were used to ensure high coverage for complementing analyses, as indicated. Platform information, raw data and detailed protocols for the microarray analysis are uploaded to and available from the Gene Expression Omnibus data repository (GSE76251; Edgar, Domrachev, & Lash, 2002). To assess overrepresented gene ontologies and enriched transcription factor networks among differentially expressed genes, the Pathway Studio software (Elsevier, Philadelphia, PA) was used, and *p* < 0.05 considered significantly enriched. Previous microarray data, available in ArrayExpress [E‐MEXP‐1809], exploring gene regulation during differentiation of these cells was used for comparisons. For qPCR, relative gene expression levels were calculated using the ${\Delta \Delta C}_{t}$ method with Student's unpaired two‐tailed *t* test, or multiple comparison one‐way analysis of variance, for significance test, with *p* < 0.05 considered significantly different between the groups. Predicted mRNA targets of miR‐206 were identified using TargetScanMouse. For all remaining experiments, the Student's unpaired two‐tailed *t* test was used and differences were considered significant if *p* < 0.05.

## 3 RESULTS

miR‐206 is known to attenuate proliferation and migration of breast cancer cells and is considered a tumor suppressor miRNA (Wang et al., 2014). Like other tumor suppressors, miR‐206 is lost in tumors. Its precise role in nontumor mammary cells has not been explored.

### 3.1 miR‐206 is upregulated in undifferentiated mammary cells in vitro and in vivo

We have previously shown that miR‐206 is upregulated in the proliferative, stem‐like stage (P) of nontumor HC11 cells and downregulated upon induced differentiation (Aydoğdu et al., 2012). We confirmed this expression pattern using alternative technology (TaqMan small RNA qPCR). As shown in Figure 1a, miR‐206 is expressed about three‐fold more in the P stage compared to the predifferentiation stage (PD), and two‐fold more than in the functionally differentiated (FD) stage. This reproduces our previous results where SYBR‐based qPCR technology was applied (Aydoğdu et al., 2012). Mid‐pregnancy mammary gland is a time in the mammary development when adult mammary stem cells undergo massive expansion before final alveolar differentiation begins. This may be considered the in vivo equivalent to the proliferative stage of HC11 cells (Williams et al., 2009). Therefore, we examined the mature miR‐206 levels in mammary gland of virgin (*n* = 4), pregnant (Day 10; *n* = 2), and lactating (Day 6; *n* = 3) mice. As shown in Figure 1b, the miR‐206 level was, in accordance with this assumption, increased in the midpregnancy gland, whereas the terminally differentiated lactating gland had again reduced levels of miR‐206. Thus, miR‐206 is increased in cells and tissues enriched for mammary stem cells.

### 3.2 The miR‐206 regulated gene landscape in undifferentiated mammary cells

miRNAs regulate genes post‐transcriptionally, in part by mediating degradation of target mRNAs. To explore the genome‐wide regulatory impact of miR‐206 in an unbiased manner, we performed microarray analysis. We transfected undifferentiated HC11 cells with miR‐206 mimic or negative control, twice in 24 hr intervals, and compared the resulting gene expression. Using a stringent cut‐off point, we identified between 285 (*p* < 0.05 and | > 0.8) differentially expressed genes (Tables S1–S2). Among the 20 most downregulated genes (Table 1), we noted its validated target *Coro1C* (Wang et al., 2014) as well as several predicted targets (*Mxd4, Tmsb4x, Sfrp1, Ptma*). For gene ontology overrepresentation analysis, we used a less strict cut‐off to capture all variations (*p* < 0.1 and | > 0.6, Figure 1c) which identified 524 differentially expressed genes (Table S1–2). This analysis indicated that the cell cycle (*p* = 4.9 × 10^−8^; Table 2) was the most enriched function among downregulated genes, and type I interferon‐mediated signaling pathway (*p *= 7.9 × 10^−10^; Table 3) among upregulated genes. We also note that various developmental functions, Wnt receptor regulation and stem‐cell maintenance were overrepresented among the miR‐206‐repressed genes. The full lists, including gene names, are provided in Tables S3‐S4. Thus, this analysis identified a miR‐206‐regulated gene landscape including numerous genes involved in proliferation.

**Table 1:** The top‐20 genes that were significantly downregulated by miR‐206 in undifferentiated mammary epithelial cells

Official gene symbol	Gene ID	logFC	*p*‐Value
S100a4	4858	−1.6	0.004
D12Ertd647e	15363	−1.6	0.004
Sfrs9	2591	−1.3	0.011
OTTMUSG00000000267	18489	−1.2	0.011
Crip1	17342	−1.3	0.012
Mxd4^a^	28181	−1.2	0.012
Nuf2	2576	−1.2	0.015
Tk1	29654	−1.3	0.015
Tmsb4x^a^	27423	−2.0	0.015
D15Bwg0580e	15774	−1.1	0.016
Coro1c^b^	921	−1.1	0.016
Fxyd5	27315	−1.1	0.017
Uhrf1	2928	−1.4	0.018
Tacstd1	18264	−1.1	0.020
Sfrp1^a^	4156	−1.0	0.021
Pcolce2	25114	−1.7	0.022
Shcbp1	10243	−1.0	0.022
Ptma^a^	17738	−1.0	0.024
Cav1	25822	−1.0	0.025
Cnih	24977	−0.9	0.026

*Note*. A negative log of fold change (logFC) indicates decreased levels in miR‐206 mimic treated HC11 cells compared to cells treated with negative control.

^a^ Predicted miR‐206 target perper TargetScanMouse (miR‐206‐3p).

^b^ Demonstrated miR‐206 target.

**Table 2:** Gene ontology overrepresentation analysis of miR‐206 downregulated genes

Enriched pathways (miR‐206 repressed genes)	*p*‐Value
Cell cycle	4.9E−08
Brain development	1.1E−05
S phase of mitotic cell cycle	1.3E−05
Negative regulation of BMP signaling pathway	1.3E−05
Olfactory bulb development	4.9E−05
Caveola assembly	5.4E−05
Face morphogenesis	8.2E−05
Substrate‐dependent cell migration, cell extension	0.00011
Actin cytoskeleton organization	0.00015
Wnt receptor signaling pathway involved in somitogenesis	0.00027
Lateral ventricle development	0.00027
Cell cycle checkpoint	0.00035
Neural tube development	0.00045
Canonical Wnt receptor signaling pathway	0.00047
Positive regulation of apoptosis	0.00049
Somatic stem cell maintenance	0.00053
G1‐S transition of mitotic cell cycle	0.00053
Positive regulation of canonical Wnt receptor signaling pathway	0.00057
In utero embryonic development	0.00063

*Note*. The 186 genes repressed by miR‐206 were analyzed for gene ontology enrichment using Pathway Studio software. The table shows selected overrepresented biological functions (full list is provided in Supporting Information data).

**Table 3:** Gene ontology overrepresentation analysis of miR‐206 upregulated genes

Enriched pathways (miR‐206 enhanced genes)	*p*‐Value
Type I interferon‐mediated signaling pathway	1.58E−19
Metabolic process	1.09E−10
cellular response to interferon‐β	9.25E−10
Apoptosis	9.85E−09
Regulation of apoptosis	1.98E−08
Response to cytokine stimulus	2.43E−08
DNA damage response, signal transduction by p53 class mediator resulting in cell cycle arrest	2.60E−08
Induction of apoptosis	5.22E−08
Defense response to protozoan	1.06E−07
Positive regulation of NF‐kappaB transcription factor activity	2.40E−07
JAK‐STAT cascade	7.03E−07
Regulation of cellular amino acid metabolic process	3.32E−06
Protein ubiquitination	8.82E−06
Cell cycle checkpoint	1.44E−05

*Note*. The 338 genes increased by miR‐206 were analyzed for gene ontology enrichment using Pathway Studio software. The table shows selected overrepresented biological functions (full list is provided in Supporting Information data).

### 3.3 miR‐206 induces G1‐S cell cycle arrest in undifferentiated HC11 mammary cells

miR‐206 is upregulated in the proliferating stage of HC11 cells (Figure 1a), our microarray and overrepresentation analysis indicated that miR‐206 strongly impacted cell proliferation (Table 2), repressing 15 genes associated with proliferation (including *Mki67* and *Mcm6*; Table S3). Further, we noted that “cell cycle checkpoint” was enriched (*p* = 1.4 × 10^−5^) among upregulated genes, such as *Cdkn2* which is an inhibitor of Cdk4/6. We confirmed the microarray data using qPCR and note that all tested genes confirmed the microarray results (Figure 1d). To test whether miR‐206 de facto reduced proliferation in HC11 cells, we introduced miR‐206 mimic to the undifferentiated proliferating stage and compared the number of viable cells to cells transfected with a control mimic. Using cell counting, we found that miR‐206 significantly decreased the cell viability (Figure 1e). BrdU staining corroborated that miR‐206 reduced proliferation (Figure 1f), cell cycle analysis using PI staining demonstrated that it induced a G0/G1 cell cycle arrest (Figure 1g). As genes associated with induction of apoptosis were also enriched among upregulated genes (*p *= 5.2 × 10^−8^; Table 3), we specifically investigated whether miR‐206 had an effect on cell apoptosis and we included cisplatin treatment as a positive control. Cell cycle analysis showed that miR‐206 did not induce a sub‐G1 peak indicative of apoptosis (Figure 1g). We also could not detect cleavage of apoptotic protein markers Caspase 3 (Casp3) and PARP (Parp1) after miR‐206 transfection (Figure 1h). Collectively, we demonstrate that miR‐206 inhibited cell proliferation of nontumor undifferentiated mammary epithelial stem‐like HC11 cells through a G1‐S cell cycle arrest, without a notable impact on apoptosis. Thus, the higher level of miR‐206 in the proliferating HC11 cells and in the mid‐pregnant mammary gland (Figure 1a,b) may function to control the rate of actively dividing cells, in line with the characteristics of a tumor suppressor.

### 3.4 A proliferative gene network is controlled by miR‐206

**Figure 2:**
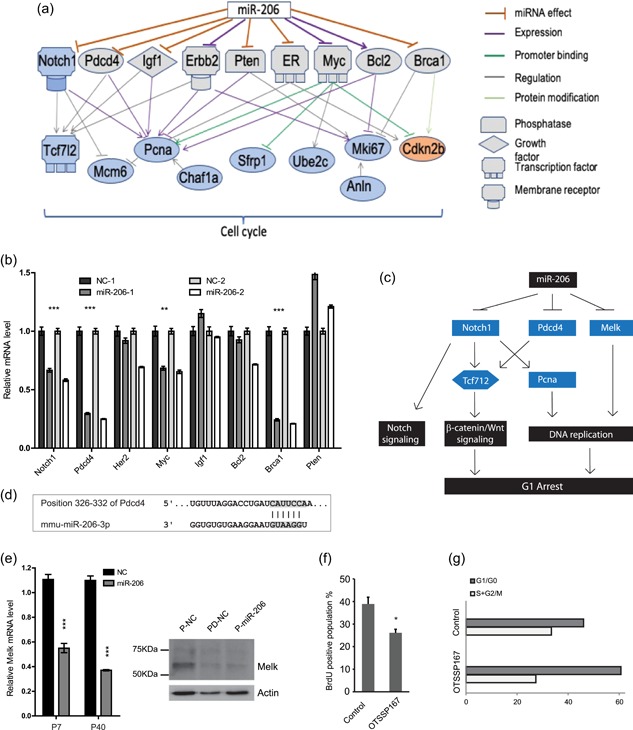
Cell cycle networks control proliferation in HC11 cells. (a) miR‐206 modulates cell cycle‐related genes through intermediators. Networks were assembled based on literature and subnetwork analysis using Pathway Studio software and our microarray data. Orange genes: upregulated by miR‐206 per our analysis; Blue genes: downregulated by miR‐206 per our analysis. (b) qPCR analysis of intermediators identified in (a) was performed, and *Notch1, Pdcd4, Myc* and *Brca1* were corroborated to be regulated by miR‐206 in HC11 cells. (c) Proposed mechanism whereby miR‐206 controls cell cycle in HC11. The pathway was assembled based on literature references and subnetwork analysis using Pathway Studio software. Blue genes: downregulated by miR‐206 (per our analyses). (d) A predicted miR‐206 target site in the 3’UTR of *Pdcd4* supports a direct mechanism. (e) Melk mRNA (left) and protein (right) are reduced by miR‐206 mimic in HC11 cells. HC11 PD stage is used as a control for lower Melk levels, as it is decreased during the differentiation process. (f) Inhibition of Melk in HC11 (P) blocks cell proliferation. Melk‐selective inhibitor (OTSSP167) reduces proliferation as quantified by BrdU staining, and (g) induces a G1–S cycle arrest. PI staining measured the G1/G0 and S+G2/M populations. Histogram bars indicate mean, +/− standard deviation (*SD*). Student's unpaired two‐tailed *t* test with significance indicated by *p < .05; **p < .01; ***p < .001. NC: negative control mimic; PI: propidium iodide; qPCR: quantitative polymerase chain reaction [Color figure can be viewed at wileyonlinelibrary.com]

To dissect how miR‐206 controls the G1‐S cell cycle arrest in these cells, we searched for direct targets among the detected downregulated proliferative genes. Utilizing software for sequence alignment analysis (TargetScan), we found that none of these genes contained predicted miR‐206‐binding sites in their 3′UTRs. This implied a possible indirect connection between miR‐206 and the proliferative genes, or that the microarray analysis had not detected all regulated genes. We examined possible theoretical intermediators: genes that according to literature could be regulated by miR‐206 and which in turn could regulate the proliferative genes we identified in our microarray. Using Pathway Studio software, we identified a number of such possible intermediators (Figure 2a). qPCR demonstrated that four (*Notch1, Pdcd4, Myc*, and *Brca1*) out of eight proposed intermediators were indeed significantly reduced by miR‐206 (Figure 2b), even though they were not identified in the microarray analysis. Of these, *Pdcd4* harbors a predicted miR‐206 target site in its 3‐UTR (Figure 2d), supporting that it could be directly repressed by miR‐206. Additionally, according to our microarray data, another gene with a prominent role in cell cycle regulation, Melk (Jiang & Zhang, 2013; Pickard et al., 2009), was repressed by miR‐206. Melk has also been reported to be upregulated in early embryonic cellular stages, in several stem‐cell populations, and in multiple types of human cancers (Jiang & Zhang, 2013; Pickard et al., 2009; Simon et al., 2017). As shown in our previous study, Melk expression is high in the undifferentiated stage of the HC11 model (Williams et al., 2009). We confirmed the miR‐206‐mediated downregulation of Melk using qPCR and western blot analysis (Figure 2e). We further explored whether Melk contributes to the miR‐206‐mediated cell cycle arrest by treating HC11 cells with Melk inhibitor OTSSP167 (Chung et al., 2013; Simon et al., 2017), followed by BrdU staining and cell cycle analysis. We found that this Melk inhibitor decreased DNA synthesis and arrested the cells in the G1 cycle (Figure 2f,g). Thus, we propose a potential model, illustrated in Figure 2c, whereby miR‐206 reduces proliferation by repressing Melk, Notch1, and Pdcd4, which then modify levels of Pcna and the Wnt‐pathway effector Tcf7I2, respectively. This pathway can contribute to reduced DNA replication, reduced β‐catenin/Wnt signaling, and G1 arrest. We conclude that our analysis identified several networks whereby miR‐206 can induce cell cycle arrest in a coordinated manner.

### 3.5 miR‐206 regulates stemness‐related markers

Among the genes that were upregulated as a result of miR‐206 addition, we note that the most overrepresented biological functions included those related to inflammatory responses (listed in Table S4), such as type I interferon‐mediated signaling pathway (*p* = 6.9 × 10^–19^ among upregulated genes), cytokine signaling (*p* = 6.9 × 10^–18^), and NFκB signaling (*p* = 2.4 × 10^−7^). These specific pathways are also connected to stem cell abilities in normal and tumor stem cells (Doherty & Jackson, 2018; Kastrati, Delgado‐Rivera, Georgieva, Thatcher, & Frasor, 2017; Morales‐Mantilla & King, 2018). Specific genes that have been identified as drivers of stemness of breast cancer stem cells (Qadir et al., 2017), such as interferon‐related developmental regulator 1 (Ifrd1) and Stat1, were among the upregulated genes (Table S2). Further, by comparing with our previous data of the HC11 stem cell‐stage transcriptome, we found that only eight of the miR‐206 mimic‐induced genes were specifically increased in the stem‐cell stage (Williams et al., 2009). Four of those genes (*Gnl3, Ifrd1, Nufip1*, and *Pawr*) are linked to stem cell abilities and/or tumorigenesis. Gln3, also called Nucleostemin, is known to enhance stemness and three‐dimensional tumor spheroid formation (Tsai & McKay, 2002). Together, this indicates that miR‐206 by inducing Ifrd1, Stat1, Gln3, Nufip1, and Pawr may have a stemness enhancing function. On the other hand, the gene ontology function “somatic stem cell maintenance” was enriched among downregulated genes (*p* = 0.0005). This group includes three genes: *Tcf7lc, Sfrp1*, and *Ski*. *Sfrp1* is a predicted target of miR‐206, and normally increases during HC11 differentiation (Williams et al., 2009). In addition, the stem cell fate‐specification gene *Cenpf* was repressed by miR‐206. The miR‐206‐mediated downregulation of *Tcf7lc, Sfrp1*, and *Cenpf* were corroborated by qPCR (Figure 1d). Thus, a number of regulations indicated that miR‐206 may either enhance or attenuate stemness.

**Figure 3:**
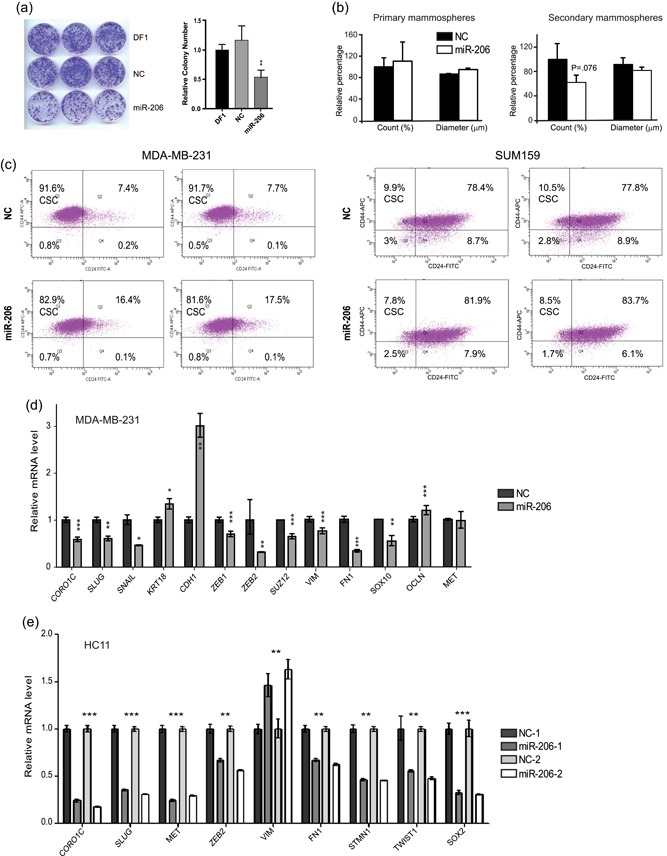
miR‐206 reduces stemness‐related markers. (a) miR‐206 reduces colony formation of HC11 cells. Pictures of colonies (10 days after miR‐206 transfection, left panel), and quantification of colony number for triplicated experiment (right panel). DF1 (Dharmafect only control), NC mimic (negative control mimic).( b) miR‐206 does not significantly affect formation or size of HC11 mammospheres. Average of three independent experiments. (c) The CD44^+^CD24^low^ population is reduced by miR‐206 in two human TNBC cell lines (*p* = .0045 and *p* = .047, respectively) compared to controls (upper panels), as quantified by measuring the CD24 and CD44 cell surface markers in MDA‐MB‐231 (left) and SUM159 (right) cells using flow cytometry. (d,e) miR‐206 reduces EMT markers in TNBC (d) and HC11 (e) cells. A series of EMT markers are measured using qPCR after addition of miR‐206 mimic. The known miR‐206 direct target, Coro1c, was used as a positive control for the assay. Histogram bars indicate mean, +/− standard deviation (SD). Student's unpaired two‐tailed *t* test with significance indicated by *p < .05; **p < .01; ***p < .001. NC: negative control mimic; qPCR: quantitative polymerase chain reaction [Color figure can be viewed at wileyonlinelibrary.com]

To test if miR‐206 modifies self‐renewal of nontumor cells, we examined the colony formation ability of undifferentiated stem‐like HC11 cells. This assay measures the capacity for unlimited proliferation of a single cell. As shown in Figure 3a, miR‐206 decreased colony formation. This assay may however be impacted by the general antiproliferative function of miR‐206. Thus, we also investigated whether miR‐206 influenced mammosphere formation of HC11 cells. The formation of primary mammospheres is used as a measure of stem cell/early progenitor activity while the formation of secondary mammospheres, generated by passaging the primary mammospheres, is used to quantify their self‐renewal (Shaw et al., 2012). After 7 days of incubation in nonadherent conditions, HC11 cells formed primary mammospheres (Figure 3b; left). miR‐206 treatment, however, did not significantly impact their number or size (Figure 3b; left). Because HC11 cells tend to aggregate and form clumps when transferred to serum‐free medium, the result of the primary mammosphere assay may be less reliable. The secondary mammosphere culture did not suffer from aggregates to the same extent. The number of secondary mammospheres did clearly not increase by miR‐206, rather they exhibited a nonsignificant decrease in (*p* = 0.076; Figure 3b; right). Several EMT markers (including *Slug, Met, Zeb2, Fn1, Twist1*, and *Sox2*) were downregulated by miR‐206 in the HC11cells (Figure 3e). *Coro1c*, a demonstrated miR‐206 target witch impacts metastasis, and Zeb2 and Fn1 are predicted miR‐206 targets. Collectively, EMT markers, colony and mammosphere formation data reveal that miR‐206 did not enhance stemness of nontumor HC11 cells but appear to reduce it.

### 3.6 miR‐206 reduces EMT markers in human TNBC cells

To investigate reduction of stemness by miR‐206 further, we transfected human mesenchymal‐like TNBC cell lines (MDA‐MB‐231 and SUM159) which express low levels of miR‐206 (Wang et al., 2014), with miR‐206 mimic and analyzed the CD44^+^CD24^low^ population and EMT markers. The CD44^+^CD24^low^ population is enriched for cancer stem cells (CSCs) although most cells in this population are not true CSCs. EMT markers are closely linked to CSC activity. We found that the CD44^+^CD24^low^ population was reduced by miR‐206 mimic treatment in both MDA‐MB‐231 and SUM159 cells (Figure 3c), and that multiple mesenchymal markers were decreased (*VIM, FN1, SLUG, SNAIL, ZEB1, ZEB2, SUZ12*) while the epithelial marker E‐cadherin (*CDH1*) was increased (Figure 3d). This supports that miR‐206 opposes EMT and corresponding CSC characteristics in human TNBC cells. We note some species or cell‐specific differences, such as that *VIM* was reduced by miR‐206 in the TNBC cells but increased in HC11 cells, and *Met* (also predicted miR‐206 target) was repressed in HC11 but not in TNBC cells. Taken together, we found support that miR‐206 decreased EMT markers in both nontumor murine HC11 cells and in human TNBC cells.

### 3.7 Epithelial differentiation and lipid accumulation are enhanced by miR‐206

**Figure 4:**
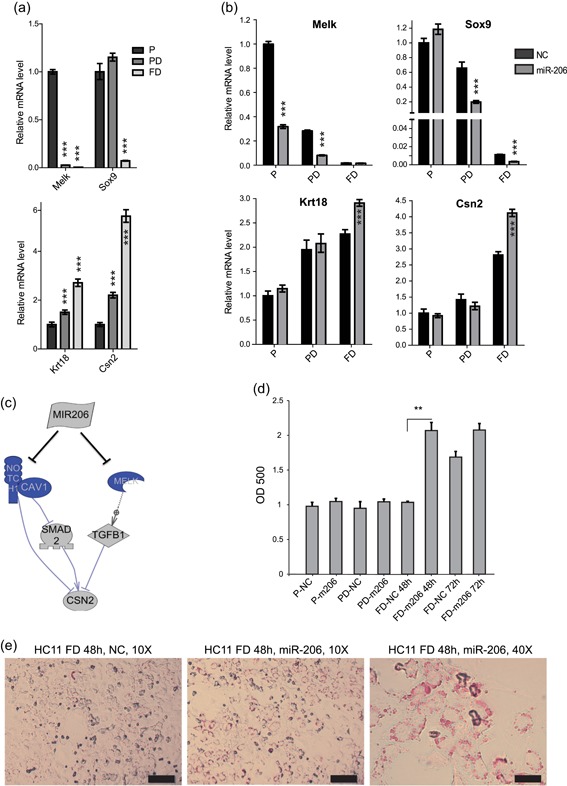
miR‐206 enhances differentiation. (a) Previously identified markers of undifferentiation (*Melk, Sox9*) and differentiation (*Krt18, Csn2*) are confirmed to be strongly regulated during the HC11 differentiation process, per qPCR. (b) HC11 cells were continuously transfected with miR‐206 mimic while induced into functionally differentiated cells (Figure S1), leading to reduced expression of mesenchymal markers (*Melk* and *Sox9*) and increased expression of mammary epithelium markers (*Krt18* and *Csn2*), examined using qPCR. (c) A proposed signaling pathways whereby miR‐206 controls mammary epithelium differentiation. Regulations are assembled based on our subnetwork analysis using Pathway Studio software coupled with literature. Blue genes: downregulated by miR‐206 per microarray/qPCR. (d,e) miR‐206 promotes lipid accumulation, a functional sign of mammary differentiation, in HC11 cells. Total lipid content is measured using Oil Red O staining, and is observed to be enhanced 48 hr after the functional differentiation in miR‐206 treated cells (d) and visualized in (e), scale bars: 100 μm for 10×, and 25 μm for 40×. Histogram bars indicate mean, +/− *SD*. Student's unpaired two‐tailed *t* test with significance indicated by *p < .05; **p < .01; ***p < .001. NC: negative control mimic; qPCR: quantitative polymerase chain reaction; SD: standard deviation [Color figure can be viewed at wileyonlinelibrary.com]

We have found that miR‐206 decreased proliferation, EMT, and stemness. To investigate if this, in turn, leads to an overall more differentiated phenotype, we first determined whether added miR‐206 led to a more differentiation‐like transcriptome. We compared the miR‐206‐induced gene expression profile with that of the transition from the stem cell‐like (P) stage to the committed predifferentiated (PD) stage in HC11 cells (Williams et al., 2009). Nearly one fifth of miR‐206 mimic‐regulated genes (94 of 534, Supp. Table 3) were also regulated during this differentiation step (Figure 1c). The majority of these were increased by miR‐206 and increased during differentiation (37 genes) or decreased by miR‐206 and decreased during differentiation (29 genes). This supports that miR‐206 leads to a more differentiation‐like transcriptome. Next, we explored weather miR‐206 indeed enhanced differentiation markers during the differentiation process. As reproduced in Figure 4a, HC11 cells that undergo functional differentiation cease to express *Melk* and *Sox9*, while increasing expression of Keratin 18 (*Krt18*) and beta‐casein (*Csn2*), as previously determined (Williams et al., 2009). Our microarray (Table S1), qPCR and western blot analyses (Figure 2e) demonstrated that miR‐206 reduced Melk in the undifferentiated stage. To investigate this effect in further detail, we explored whether continuous miR‐206 treatment during the differentiation process affected any of those four markers. We treated HC11 cells sequentially during the differentiation process (one miR‐206 mimic transfection in each stage, see treatment layout in Figure S1), and assessed the regulations of *Melk, Sox9, Krt18* , and *Csn2* in each stage (P, PD, DIF). Again, we found that the undifferentiation marker *Melk* was reduced by miR‐206 in the P stage, and also noted significant decrease in the PD stage, while its expression had ceased in the DIF stage with or without added miR‐206 (Figure 4b, upper left panel). Similarly, *Sox9* were reduced by miR‐206, in the two later stages (Figure 4b, upper right panel). On the contrary, the differentiation markers (*Krt18* and *Csn2*) were enhanced by miR‐206, although only in the fully differentiated stage (Figure 4b, lower panel). Using microarray and Pathway Studio software, we attempted to propose a model for how miR‐206 upregulates the key differentiation marker beta casein (Csn2). This model suggests that repression of *Melk, Notch1*, and *Cav1* (all detected in the microarray), enhance Smad2 activity and reduce Tgfb1. As Smad2 enhances and Tgfb1 decreases Csn2, these regulations lead to increased beta‐casein expression (Figure 4c).

To functionally investigate whether differentiation is indeed enhanced, we examined the lipid content of the treated HC11 cells. Lipid accumulation is a hallmark of the mammary gland differentiation (Rudolph et al., 2007), and we have previously demonstrated that lipogenesis is increased during HC11 differentiation (Doria et al., 2014). Our microarray data indicated that lipid‐associated genes were regulated by miR‐206 (*Apod, Acadl, Trib3, Arf14, Cxcl16, Fhl2*, and *Ankrd1* were upregulated; *Gpd2, Lrp6*, and *Pccb* downregulated). We measured the lipid content of HC11 cells after treatment with miR‐206 mimic or negative control during the differentiation process, using Oil Red O staining. We found that transfection of miR‐206 mimic enhanced the lipid accumulation, generating a maximal amount of lipid droplets already 48 hr after functional differentiation was initiated, compared to the 72 hr required for the control mimic (Figure 4d, with representative images in Figure 4e). Our interpretation is that the differentiation and corresponding ability for lipid accumulation are accelerated by miR‐206. Overall, our analyses demonstrate that miR‐206 can promote mammary cell differentiation and corresponding lipogenesis.

## 4 DISCUSSION

miR‐206 impacts the development of the mammary gland, and is considered to be a tumor suppressor which is lost in breast cancer. However, suppression of tumor development primarily takes in the normal cell, to protect it from tumorigenesis, and exactly how miR‐206 mediates its developmental and tumor suppressive functions in normal mammary cells is not known. Our study aimed to elucidate this and gain insights into its precise activities during mammary gland development, to help delineate its tumor suppressive functions.

Our results demonstrate that miR‐206 regulates several important pathways and functions in nontumor mammary cells, which can help suppress tumor development. We found that miR‐206 modulates the expression of several hundreds of genes, and that this impacts three important functions: cell cycle (G1 arrest), stemness (control), and differentiation (enhancement). Our data agree with the recent findings that miR‐206 reduces breast CSC (Samaeekia et al., 2017). We deduce that the upregulation of miR‐206 in the stem‐like stage and in the mammary gland during pregnancy functions to control cell proliferation and stemness, as well as enable differentiation, and that this is a key part of its tumor suppressor role. The upregulation in the pregnant mammary gland is based on data from only from two mice, thus, this result should be replicated in more studies, along with in situ hybridization to identify the precise cells that express this miRNA. Our finding that miR‐206 induced differentiation of HC11 cells is in line with functions that miR‐206 has in muscles. miR‐206 was first reported to be a muscle‐specific miRNA and a critical factor in muscle development (Anderson, Catoe, & Werner, 2006; Kim, Lee, Sivaprasad, Malhotra, & Dutta, 2006; Rao, Kumar, Farkhondeh, Baskerville, & Lodish, 2006), where it can induce myoblast differentiation by modulation of the Wnt signaling pathway (Dey, Gagan, & Dutta, 2010; Winbanks et al., 2011). Our data thus suggest that its role as a differentiation factor is conserved between muscle and mammary gland. Similar to in breast cancer, miR‐206 is lost in myosarcoma (Missiaglia et al., 2010) and its re‐expression can block myosarcoma growth by inducing differentiation (Mishra & Merlino, 2009; Taulli et al., 2009).

The strengths of our study include that we used an unbiased and comprehensive approach to discover the networks regulated by miR‐206 (microarray), that we used nontumor cells to explore its normal role, and that we combined the analysis with functional studies, including differentiation capacity. Limitations of our study include that we used only one cell line for most of the studies. The rational was to provide a comprehensive “map” of functions that can be regulated by miR‐206 in nontumor mammary cells, and it will be important that our results are corroborated by other models, including in vivo models, in the future. Further, we have primarily measured mRNA levels and applying measurement of protein levels, for example by employing mass spectrometry, would likely uncover additional regulations that are mediated by miR‐206. Our experimental approach did not test for opposite activities using an miR‐206 inhibitor. Therefore, all results presented here should be viewed with these aspects in mind.

Overall, we propose key roles for miR‐206 during mammary gland development, and we also delineate multiple beneficial impacts that reintroduction of miR‐206 into breast cancer cells can have.

## CONFLICT OF INTERESTS

The authors do not declare any conflict of interest.

## DATA AVAILABILITY

The data that support the findings of this study are openly available at the Gene Expression Omnibus data repository at https://www.ncbi.nlm.nih.gov/geo/, reference number [GSE76251].

## Supporting information

Supporting information

Supporting information

Supporting information

## References

[jcp28789-bib-0001] Adams, B. D. , Furneaux, H. , & White, B. A. (2007). The micro‐ribonucleic acid (miRNA) miR‐206 targets the human estrogen receptor‐alpha (ERalpha) and represses ERalpha messenger RNA and protein expression in breast cancer cell lines. Molecular Endocrinology, 21(5), 1132–1147. doi:me.2007‐0022 [pii] 10.1210/me.2007‐0022 17312270 10.1210/me.2007-0022

[jcp28789-bib-0002] Anderson, C. , Catoe, H. , & Werner, R. (2006). MIR‐206 regulates connexin43 expression during skeletal muscle development. Nucleic Acids Research, 34(20), 5863–5871. doi:gkl743 [pii] 10.1093/nar/gkl743 17062625 10.1093/nar/gkl743 PMC1635318

[jcp28789-bib-0003] Aydoğdu, E. , Katchy, A. , Tsouko, E. , Lin, C. Y. , Haldosen, L. A. , Helguero, L. , … Williams, C. (2012). MicroRNA‐regulated gene networks during mammary cell differentiation are associated with breast cancer. Carcinogenesis, 33(8), 1502–1511. doi:bgs161 [pii] 10.1093/carcin/bgs161 22562546 10.1093/carcin/bgs161

[jcp28789-bib-0004] Ball, R. K. , Friis, R. R. , Schoenenberger, C. A. , Doppler, W. , & Groner, B. (1988). Prolactin regulation of beta‐casein gene expression and of a cytosolic 120‐kd protein in a cloned mouse mammary epithelial cell line. EMBO Journal, 7(7), 2089–2095. 3416834 10.1002/j.1460-2075.1988.tb03048.x PMC454494

[jcp28789-bib-0005] Chung, S. , Suzuki, H. , Miyamoto, T. , Takamatsu, N. , Tatsuguchi, A. , Ueda, K. , … Matsuo, Y. (2013). Development of an orally‐administrative MELK‐targeting inhibitor that suppresses the growth of various types of human cancer. Oncotarget, 3(12), 1629–1640. doi:790 [pii] 10.18632/oncotarget.790 PMC3681500 23283305

[jcp28789-bib-0006] Dey, B. K. , Gagan, J. , & Dutta, A. (2010). miR‐206 and ‐486 induce myoblast differentiation by downregulating Pax7. Molecular and Cellular Biology, 31(1), 203–214. doi:MCB.01009‐10 [pii] 10.1128/MCB.01009‐10 21041476 10.1128/MCB.01009-10 PMC3019853

[jcp28789-bib-0007] Doherty, M. R. , & Jackson, M. W. (2018). The Critical, Clinical Role of Interferon‐Beta in Regulating Cancer Stem Cell Properties in Triple‐Negative Breast Cancer. DNA and Cell Biology, 37(6), 513–516. 10.1089/dna.2018.4247 29750542 PMC5985904

[jcp28789-bib-0008] Doria, M. L. , Ribeiro, A. S. , Wang, J. , Cotrim, C. Z. , Domingues, P. , Williams, C. , … Helguero, L. A. (2014). Lipid metabolism changes throughout differentiation of mammary epithelial cells. The FASEB Journal, 28, 4247–4264. 24970396 10.1096/fj.14-249672

[jcp28789-bib-0009] Edgar, R. , Domrachev, M. , & Lash, A. E. (2002). Gene Expression Omnibus: NCBI gene expression and hybridization array data repository. Nucleic Acids Research, 30(1), 207–210. 11752295 10.1093/nar/30.1.207 PMC99122

[jcp28789-bib-0010] Edvardsson, K. , Ström, A. , Jonsson, P. , Gustafsson, J. ‐Å. , & Williams, C. (2011). Estrogen receptor beta induces anti‐inflammatory and anti‐tumorigenic networks in colon cancer cells. Molecular Endocrinology, 25(6), 969–979. 21493669 10.1210/me.2010-0452 PMC5417254

[jcp28789-bib-0011] Hesari, Z. , Nourbakhsh, M. , Hosseinkhani, S. , Abdolvahabi, Z. , Alipour, M. , Tavakoli‐Yaraki, M. , … Yarahmadi, S. (2018). Down‐regulation of NAMPT expression by mir‐206 reduces cell survival of breast cancer cells. Gene, 673, 149–158. 10.1016/j.gene.2018.06.021 29886033

[jcp28789-bib-0012] Iliopoulos, D. , Lindahl‐Allen, M. , Polytarchou, C. , Hirsch, H. A. , Tsichlis, P. N. , & Struhl, K. (2010). Loss of miR‐200 inhibition of Suz12 leads to polycomb‐mediated repression required for the formation and maintenance of cancer stem cells. Molecular Cell, 39(5), 761–772. 20832727 10.1016/j.molcel.2010.08.013 PMC2938080

[jcp28789-bib-0013] Jiang, P. , & Zhang, D. (2013). Maternal embryonic leucine zipper kinase (MELK): A novel regulator in cell cycle control, embryonic development, and cancer. International Journal of Molecular Sciences, 14(11), 21551–21560. doi:ijms141121551 [pii] 10.3390/ijms141121551 24185907 10.3390/ijms141121551 PMC3856021

[jcp28789-bib-0014] Kastrati, I. , Delgado‐Rivera, L. , Georgieva, G. , Thatcher, G. R. , & Frasor, J. (2017). Synthesis and characterization of an aspirin‐fumarate prodrug that inhibits NFkappaB activity and breast cancer stem cells. Journal of Visualized Experiments: JoVE, (119) 10.3791/54798 PMC5352264 28190074

[jcp28789-bib-0015] Kim, H. K. , Lee, Y. S. , Sivaprasad, U. , Malhotra, A. , & Dutta, A. (2006). Muscle‐specific microRNA miR‐206 promotes muscle differentiation. Journal of Cell Biology, 174(5), 677–687. doi:jcb.200603008 [pii] 10.1083/jcb.200603008 16923828 10.1083/jcb.200603008 PMC2064311

[jcp28789-bib-0016] Jung, M. J. , Yoon, K. S. , Cho, K. W. , Kim, K. S. , & Jung, H. S. Expression of miR‐206 during the initiation of mammary gland development. Cell and Tissue Research, 353(3), 425–433. 23733266 10.1007/s00441-013-1653-3

[jcp28789-bib-0017] Li, Y. , Hong, F. , & Yu, Z. (2013). Decreased expression of microRNA‐206 in breast cancer and its association with disease characteristics and patient survival. Journal of International Medical Research, 41(3), 596–602. doi:0300060513485856 [pii] 10.1177/0300060513485856 23696595 10.1177/0300060513485856

[jcp28789-bib-0018] Merlo, G. R. , Graus‐Porta, D. , Cella, N. , Marte, B. M. , Taverna, D. , & Hynes, N. E. (1996). Growth, differentiation and survival of HC11 mammary epithelial cells: Diverse effects of receptor tyrosine kinase‐activating peptide growth factors. European Journal of Cell Biology, 70(2), 97–105. 8793381

[jcp28789-bib-0019] Mishra, P. J. , & Merlino, G. (2009). MicroRNA reexpression as differentiation therapy in cancer. Journal of Clinical Investigation, 119(8), 2119–2123. doi:40107 [pii] 10.1172/JCI40107 19620782 10.1172/JCI40107 PMC2719926

[jcp28789-bib-0020] Missiaglia, E. , Shepherd, C. J. , Patel, S. , Thway, K. , Pierron, G. , Pritchard‐Jones, K. , … Shipley, J. (2010). MicroRNA‐206 expression levels correlate with clinical behaviour of rhabdomyosarcomas. British Journal of Cancer, 102(12), 1769–1777. doi:6605684 [pii] 10.1038/sj.bjc.6605684 20502458 10.1038/sj.bjc.6605684 PMC2883695

[jcp28789-bib-0021] Monteiro, F. L. , Vitorino, R. , Wang, J. , Cardoso, H. , Laranjeira, H. , Simões, J. , … Helguero, L. A. (2017). The histone H2A isoform Hist2h2ac is a novel regulator of proliferation and epithelial‐mesenchymal transition in mammary epithelial and in breast cancer cells. Cancer Letters, 296, 42–52. 10.1016/j.canlet.2017.03.007 28288875

[jcp28789-bib-0022] Morales‐Mantilla, D. E. , & King, K. Y. (2018). The role of interferon‐gamma in hematopoietic stem cell development, homeostasis, and disease. Current Stem Cell Reports, 4(3), 264–271. 10.1007/s40778-018-0139-3 30148048 PMC6096761

[jcp28789-bib-0023] Nguyen, L. V. , Vanner, R. , Dirks, P. , & Eaves, C. J. (2012). Cancer stem cells: An evolving concept. Nature Reviews Cancer, 12(2), 133–143. 10.1038/nrc3184 22237392

[jcp28789-bib-0024] Pickard, M. R. , Green, A. R. , Ellis, I. O. , Caldas, C. , Hedge, V. L. , Mourtada‐Maarabouni, M. , … Williams, G. T. (2009). Dysregulated expression of Fau and MELK is associated with poor prognosis in breast cancer. Breast Cancer Research, 11(4), R60. doi:bcr2350 [pii] 10.1186/bcr2350 19671159 10.1186/bcr2350 PMC2750122

[jcp28789-bib-0025] Qadir, A. S. , Ceppi, P. , Brockway, S. , Law, C. , Mu, L. , Khodarev, N. N. , … Peter, M. E. (2017). CD95/Fas increases stemness in cancer cells by inducing a STAT1‐dependent type i interferon response. Cell Reports, 18(10), 2373–2386. 10.1016/j.celrep.2017.02.037 28273453 PMC5474321

[jcp28789-bib-0026] Rao, P. K. , Kumar, R. M. , Farkhondeh, M. , Baskerville, S. , & Lodish, H. F. (2006). Myogenic factors that regulate expression of muscle‐specific microRNAs. Proceedings of the National Academy of Sciences of the United States of America, 103(23), 8721–8726. doi:0602831103 [pii] 10.1073/pnas.0602831103 16731620 10.1073/pnas.0602831103 PMC1482645

[jcp28789-bib-0027] Rudolph, M. C. , McManaman, J. L. , Phang, T. , Russell, T. , Kominsky, D. J. , Serkova, N. J. , … Neville, M. C. (2007). Metabolic regulation in the lactating mammary gland: A lipid synthesizing machine. Physiological Genomics, 28(3), 323–336. 10.1152/physiolgenomics.00020.2006 17105756

[jcp28789-bib-0028] Samaeekia, R. , Adorno‐Cruz, V. , Bockhorn, J. , Chang, Y. F. , Huang, S. , Prat, A. , … Liu, H. (2017). miR‐206 inhibits stemness and metastasis of breast cancer by targeting MKL1/IL11 pathway. Clinical Cancer Research, 23(4), 1091–1103. 10.1158/1078-0432.Ccr-16-0943 27435395 PMC5247402

[jcp28789-bib-0029] Shaw, F. L. , Harrison, H. , Spence, K. , Ablett, M. P. , Simoes, B. M. , Farnie, G. , … Clarke, R. B. (2012). A detailed mammosphere assay protocol for the quantification of breast stem cell activity. Journal of Mammary Gland Biology and Neoplasia, 17(2), 111–117. 10.1007/s10911-012-9255-3 22665270

[jcp28789-bib-0030] Shimono, Y. , Zabala, M. , Cho, R. W. , Lobo, N. , Dalerba, P. , Qian, D. , … Clarke, M. F. (2009). Downregulation of miRNA‐200c links breast cancer stem cells with normal. Stem Cells, 138(3), 592–603. 10.1016/j.cell.2009.07.011 PMC2731699 19665978

[jcp28789-bib-0031] Simon, M. , Mesmar, F. , Helguero, L. , & Williams, C. (2017). Genome‐wide effects of MELK‐inhibitor in triple‐negative breast cancer cells indicate context‐dependent response with p53 as a key determinant. PLoS One, 12(2), e0172832 10.1371/journal.pone.0172832 28235006 PMC5325553

[jcp28789-bib-0032] Taulli, R. , Bersani, F. , Foglizzo, V. , Linari, A. , Vigna, E. , Ladanyi, M. , … Ponzetto, C. (2009). The muscle‐specific microRNA miR‐206 blocks human rhabdomyosarcoma growth in xenotransplanted mice by promoting myogenic differentiation. Journal of Clinical Investigation, 119(8), 2366–2378. doi:38075 [pii] 10.1172/JCI38075 19620785 10.1172/JCI38075 PMC2719932

[jcp28789-bib-0033] Tsai, R. Y. , & McKay, R. D. (2002). A nucleolar mechanism controlling cell proliferation in stem cells and cancer cells. Genes and Development, 16(23), 2991–3003. 12464630 10.1101/gad.55671 PMC187487

[jcp28789-bib-0034] Tsouko, E. , Wang, J. , Frigo, D. E. , Aydoğdu, E. , & Williams, C. (2015). miR‐200a inhibits migration of triple‐negative breast cancer cells through direct repression of the EPHA2 oncogene. Carcinogenesis, 36(9), 1051–1060. 26088362 10.1093/carcin/bgv087 PMC5975720

[jcp28789-bib-0035] Vahidian, F. , Mohammadi, H. , Ali‐Hasanzadeh, M. , Derakhshani, A. , Mostaan, M. , Hemmatzadeh, M. , … Baradaran, B. (2018). MicroRNAs and breast cancer stem cells: Potential role in breast cancer therapy. Journal of Cellular Physiology, 234, 3294–3306. 10.1002/jcp.27246 30362508

[jcp28789-bib-0036] Wang, J. , Tsouko, E. , Jonsson, P. , Bergh, J. , Hartman, J. , Aydoğdu, E. , & Williams, C. (2014). miR‐206 inhibits cell migration through direct targeting of the actin‐binding protein coronin 1C in triple‐negative breast cancer. Molecular oncology, 8(8), 1690–1702. 10.1016/j.molonc.2014.07.006 25074552 PMC5528580

[jcp28789-bib-0037] Williams, C. , Helguero, L. , Edvardsson, K. , Haldosen, L. A. , & Gustafsson, J. A. (2009). Gene expression in murine mammary epithelial stem cell‐like cells shows similarities to human breast cancer gene expression. Breast Cancer Research, 11(3), R26. doi:bcr2256 [pii] 10.1186/bcr2256 [doi] 19426500 10.1186/bcr2256 PMC2716494

[jcp28789-bib-0038] Winbanks, C. E. , Wang, B. , Beyer, C. , Koh, P. , White, L. , Kantharidis, P. , … Gregorevic, P. (2011). TGF‐beta regulates miR‐206 and miR‐29 to control myogenic differentiation through regulation of HDAC4. Journal of Biological Chemistry, 286(16), 13805–13814. doi:M110.192625 [pii] 10.1074/jbc.M110.192625 21324893 10.1074/jbc.M110.192625 PMC3077581

[jcp28789-bib-0039] Wronski, A. , Sandhu, G. K. , Milevskiy, M. J. , Brewster, B. L. , Bridge, J. A. , Shewan, A. M. , … Brown, M. A. (2016). MicroRNA‐206 is differentially expressed in Brca1‐deficient mice and regulates epithelial and stromal cell compartments of the mouse mammary gland. Oncogenesis, 5, e218–e218. 10.1038/oncsis.2016.27 27043663 PMC4848838

