# Supplementary material for: A miR‐206 regulated gene landscape enhances mammary epithelial differentiation
Source: J Cell Physiol. 2019 May 8;234(12):22220–33. doi: 10.1002/jcp.28789 (PMC6767383; doi:10.1002/jcp.28789)
Supplement: Supplementary file 1 — Supporting information [file JCP-234-22220-s001.pdf]

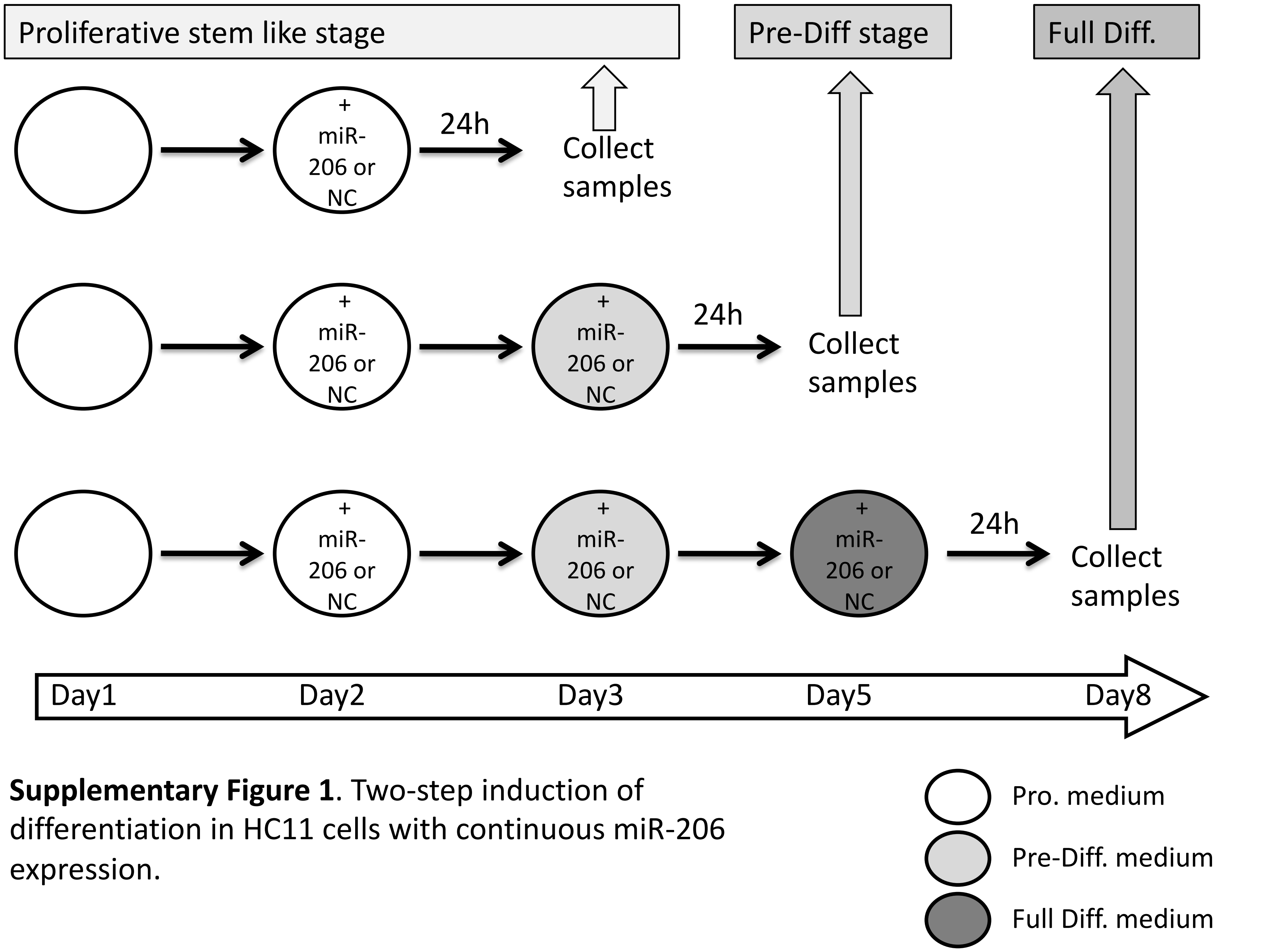

Supplementary Figure 1. Two-step induction of differentiation in HC11 cells with continuous miR-206 expression.
