# Supplementary material for: A miR‐206 regulated gene landscape enhances mammary epithelial differentiation
Source: J Cell Physiol. 2019 May 8;234(12):22220–33. doi: 10.1002/jcp.28789 (PMC6767383; doi:10.1002/jcp.28789)
Supplement: Supplementary file 3 — Supporting information [file JCP-234-22220-s003.docx]

SUPPLEMENTARY FILES

**Supplementary Figure S1**. Lay out for two-step induction of differentiation in HC11 cells with continuous miR-206 expression.

**Supplementary Table S1**: Genes downregulated by miR-206 in HC11 (P) stage, per microarray analysis. Table includes Official gene symbol, Gene ID, log of fold change (logFC), p-value, and separate data for replicated microarray experiments (Array1 and Array2). Blue font indicates qPCR corroborations.

**Supplementary Table S2**: Genes upregulated by miR-206 in HC11 (P) stage, per microarray analysis. Table includes Official gene symbol, Gene ID, log of fold change (logFC), p-value, and separate data for replicated microarray experiments (Array1 and Array2). Blue font indicates qPCR corroborations.

**Supplementary Table S3:** Enriched biological functions among genes downregulated by miR-206 in HC11 (P) stage, per microarray analysis. Table includes p-value for enrichment analysis (Pathway Studio), and official gene symbol of regulated genes. qPCR corroborations.

**Supplementary Table S4:** Enriched biological functions among genes upregulated by miR-206 in HC11 (P) stage, per microarray analysis. Table includes p-value for enrichment analysis (Pathway Studio), and official gene symbol of regulated genes. qPCR corroborations.

**Supplementary Table S5:** Genes that are both regulated by miR-206 in HC11 (P) stage, and regulated during differentiation of HC11 cells (P to PD stage), per microarray analysis. Table includes Official gene symbol, Gene ID, log of fold change (logFC) for miR-206 regulation and corresponding p-value, and log of fold change for differentiation regulation (all significant). Red color indicate upregulation, green downregulation.
